# The Institutional Library

**Published:** 1920-11-06

**Authors:** 


### THE INSTITUTIONAL LIBRARY.

**Infectious Diseases.** By C. B. Ker, M.D., F.R.C.P.Ed. 2nd Edition. (Oxford Medical Publications: Frowde, and Hodder & Stoughton. Pp. 627. Price £2 2s. net.)

This sumptuous volume is the last word, for the moment, on the subject of which it treats—the infectious fevers of this country. It is informed throughout by the wide clinical experience of the author, who strikes the personal note of actual clinical knowledge in preference to the quoting of large numbers of authorities. While this plan makes his book exceptionally useful to the man who is treating a case and wants guidance, it does deprive it of some of its value to the man who is doing research and hopes to find an exhaustive bibliography on some particular infectious fever. Dr. Ker is cautious in quoting, or at least in adopting, claims which he has not himself been able to test: he is evidently, and so far rightly, sceptical about the value of vaccine treatment of those acute fevers whose organisms have been identified. The chapter on fever-hospital problems contains discussions of much interest on such topics as the co-existence of more than one infectious disease; the question of the merits of "cubicle," "barrier," and "bed" isolations respectively; cross-infections; "return cases"; and the aggravation of disease by super-added septic complications.

There are some superb coloured photographs illustrating certain rashes: most of the plates are, however, ordinary photographs, which, though good in themselves, suffer necessarily by comparison with the coloured ones. Print, paper, and format are alike excellent; the enterprising firm which publishes these Oxford Medical Publications is entitled to great credit for the way in which Dr. Ker's valuable textbook has been produced. The whole volume reflects credit on the author and the British school of medicine: it is eminently thorough and practical and free from crankiness, and will be found of immense help by all those who may be called upon to treat cases of the infectious fevers.

**Sterile Marriages.** By J. Dulberg, M.D. (T. Werner Laurie. 1920. Pp. 264. Price 6s. net.)

This book sets out for the instruction of married men and women not merely the causes and cure of sterile marriage, but also a great variety of information on sexual affairs. The author writes with great assurance, and in a tone of authority, but, though most of what he has to say is true enough, he has fallen into three or four really serious errors, which make it difficult to recommend his book to the general public.

**Intestinal Disinfection.** By J. T. Ainslie Walker.

This little brochure of about a dozen pages devotes the first half of its extent to a review of some of the present known intestinal antiseptics, and discusses their efficiency. We are then shown the new intestinal antiseptics—trimethol and dimethol—which are phenol derivatives, both with very high Rideal Walker coefficients. They seem remarkable compounds, because very large doses can be administered with apparently no untoward results.

As modern and enlightened research attributes so many of the ills of mankind to alimentary toxæmia, we shall welcome any intestinal antiseptic that can stand the test of repeated trials and live up to its reputation by reducing alimentary sepsis.

**Radiant Motherhood.** By Marie Stopes, D.Sc. (Putnam. Price 6s. net.)

**Wedded Love or Married Misery.** By W. N. Willis. (Anglo-Eastern Publishing Co. Price 6s. 6d. net.)

Until quite the last few years that racial prudery or hypocrisy which foreigners tell us (probably correctly) is one of our marked and most unpleasing British traits, has done us much harm by proscribing the discussion in books and in the press of many sexual and marital topics on which it is important that right and clean thinking shall prevail. This reproach the last dozen years have done much to remove; a flood of literature on these once forbidden subjects has been let loose, sometimes in a torrent which has swept garbage along in its eddies. The problem of the day is not the provision of sex instruction, but the provision of right instruction: the motives of those who seek to meet the modern demand for this knowledge may be mostly good, but their discretion and capacity are not always equal to their enthusiasm.

Mrs. Stopes has for some time been foremost as an expositor of love and marriage. She holds views which are fiercely combated by the Churches; she falls into grotesque errors now and then over technical points in medicine and obstetrics; she has a distinctly neurotic outlook; and she exaggerates woefully. But she is intensely in earnest; she is intensely reverent, even devout in handling the matters she has very strongly at heart; she has many novel observations to offer; and she possesses the confidence of very many women of the most highly educated and intellectual types. Her writings are full of interest, though readers should remember that her doctorate is not in medicine, and that many of her hypotheses are merely speculative at present. Frankly, the book is not so good as some of her previous writings: there is more to reject, less to accept, than there was in "Married Love," for instance. Still, she deserves credit for giving of the best that is in her to advance her ideals.

Mr. Willis, who appears to be himself the Anglo-Eastern Publishing Co., is a bitter opponent of Mrs. Stopes, in whom he can perceive nothing good. He certainly puts his finger on one or two of her less sensible utterances, but could make his attack much more telling if he possessed sufficient medical knowledge to correct her technical mistakes. Mr. Willis himself, however, is more open to criticism than Mrs. Stopes. His errors are more numerous, his views less original, his mind less scientific, than those of the writer he attacks. The broad sheet in which he advertises his book is drawn up in very bad taste, apparently with intentions more commercial than idealistic. His motives may possibly be good, and he certainly seems to be very earnest, but his execution is clumsy; and we cannot recommend his book as a valuable contribution to the literature of love and marriage.

**Tuberculosis.** By Dr. G. Norman Meachen. The Scientific Press, Ltd., Southampton Street, W.C. 2.

This little book is another in the series of medical handbooks published by the Scientific Press, Ltd. The subject is treated very fully and is simply expressed; in fact, in some places, some of the expressions are conversational. As a manual for nurses and health visitors, we think the book should be very useful.

